# Identification and elemental mapping of enamel minerals with electron energy-loss spectroscopy†

**DOI:** 10.1039/d4ra08124b

**Published:** 2025-05-07

**Authors:** Ya-Hsiang Hsu, Asra Hassan, Amanda Trout, John D. Bartlett, Charles E. Smith, David W. McComb

**Affiliations:** a Department of Materials Science and Engineering, The Ohio State University Columbus OH USA mccomb.29@osu.edu; b Center for Electron Microscopy and Analysis, The Ohio State University Columbus OH USA; c Division of Biosciences, The Ohio State University College of Dentistry Columbus OH USA; d Department of Biologic and Materials Sciences, University of Michigan School of Dentistry Ann Arbor MI USA; e Department of Anatomy and Cell Biology, Faculty of Medicine and Health Sciences, McGill University Montreal QC Canada

## Abstract

The identification and differentiation of the mineral phases, hydroxyapatite (HA, Ca_10_(PO_4_)_6_(OH)_2_) and octacalcium phosphate (OCP, Ca_8_H_2_(PO_4_)_6_), remains challenging because of their similar composition and chemical structure. In this research, electron energy-loss spectroscopy (EELS) analyses revealed indicators to distinguish HA and OCP and these were applied to examine mineral development in enamel from mouse incisors. Reference EELS data for HA and OCP was established with commercial HA and synthesized OCP. An evaluation of electron damage and a mitigation strategy of multipass imaging was conducted, and the electron dose limitation of OCP was identified. New insights into the mechanism of electron beam damage on the apatite crystal were obtained. With the characterization of the energy-loss spectra and the EELS simulation, the oxygen K-edge was found to be one of the indicators for the differentiation of HA from OCP. The second indicator, the Ca/P ratio, was calculated with a calibrated experimental factor of *K*_exp_. Elemental mapping was done to establish the different Ca/P ratio of HA and OCP, and the boundary between these mineral forms. EELS analysis was performed on developing enamel in wild-type (WT) and *Mmp20* knockout (KO) mice. This research establishes a protocol for EELS analysis on biological specimens and demonstrates the power and potential of EELS in biomaterial characterization.

## Abstract 2

Investigation and characterization of mineral phases in dental enamel using electron energy loss spectroscopy.

## Graphical abstract

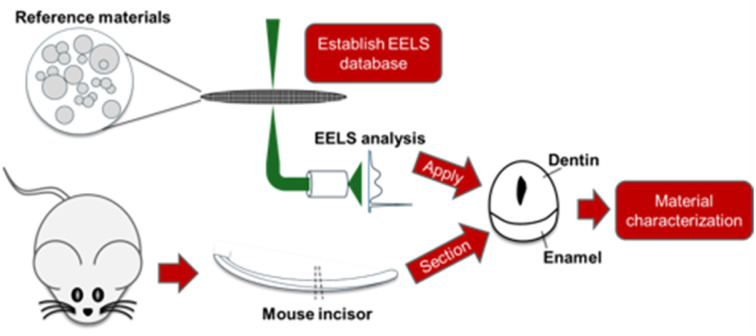

## 1. Introduction

Teeth are one of the hardest organs in the human body. The outermost enamel layer protects teeth from etching and corrosion. Typically, mature enamel is composed of 5% organic components and up to 95% minerals, with hydroxyapatite (HA, Ca_10_(PO_4_)_6_(OH)_2_) as the major constituent.^1–4^ Consequently, much of the research on tooth formation revolves around the study of HA.

The formation of enamel can be divided into three stages; the pre-secretory stage, the secretory stage, and the maturation stage.^5–7^ In the secretory stage, three enamel matrix proteins (enamelin, ameloblastin, and amelogenin) are produced to initiate the formation of enamel minerals.^8–11^ Following the emergence of enamel matrix protein, matrix metalloproteinase 20 (MMP20) is secreted to cleave proteins into fragments and is necessary for sound enamel development.^12–14^ The immature enamel has a low degree of crystallization.^4,15^ In the maturation stage, the enamel matrix proteins are cleaved into smaller fragments and absorbed by ameloblast, and the enamel minerals are fully crystalized and form the hard outer layer of teeth.^7,16,17^ Although the structure of enamel development is broadly understood, some of the details about the formation of HA minerals are still debated, such as the precise function of each enamel matrix protein and the scope of MMP20 activity.

It has been recently reported that a large portion of octacalcium phosphate (OCP, Ca_8_H_2_(PO_4_)_6_) can be detected in the enamel of the amelogenin and *Mmp20* knockout mice. Although OCP is considered to be a potential precursor in the formation of HA, it is rarely detected in normal enamel development.^5,18,19^ In 2016, an X-ray diffraction (XRD) study identified OCP crystal in the amelogenin knockout enamel.^20^ Subsequently, Yamazaki *et al.* used Raman microspectroscopy to identify a strong OCP signal in the *Mmp20* knockout enamel.^21^ More recently, a layer of fan-shaped crystals was observed to dominate the *Mmp20* knockout enamel.^14^ Using selected area electron diffraction (SAED), the authors identified the fan-shaped crystals as crystalline OCP rather than HA. However, the distinction between HA and OCP is challenging due to their chemical and crystal structure similarities. While XRD and Raman analysis can be used to identify chemical structure and crystal structure, these methods do not provide the spatial resolution to investigate the distribution of the minerals. SAED can provide crystal structure with spatial resolution but lacks chemical and elemental specificity. Therefore, a more comprehensive analytical technique is needed to identify, distinguish, and spatially map the HA and OCP phases. Electron energy-loss spectroscopy (EELS) performed in the scanning transmission electron microscope (STEM) has the potential to address this need.

EELS is used to measure the distribution of the energy-loss of primary electrons as a result of inelastic collisions with the atoms in specimens.^22,23^ The energy-loss spectrum provides information about the chemistry, electronic structure, and local bonding environment in the material.^22^ For example, Colby *et al.* investigated a range of polymers using the carbon K-edge energy-loss spectra.^24^ It was shown that the peaks in the C K-edge spectra correlate with the different functional groups in the polymer. Furthermore, they evaluated the electron beam damage on polymers based on the changes in the peaks. Finally, it was demonstrated that it is possible to map the distribution of these functional groups in polymer mixtures to obtain spatially resolved information on chemistry and bonding.

While EELS is a powerful approach to studying the local chemistry environments, the electron beam sensitivity of specimens can be a limitation to the EELS characterization. The critical electron dose not only limits the resolution of images but can induce unwanted phase transformation in the specimens.^25–27^ Xin *et al.* reported that OCP is very sensitive to the electron beam.^28^ They observed bubble-like defects after being exposed to the electron beam, and the composition slightly changed in the regions of the defects. Similar defects were also observed by Simon *et al.* with high-resolution TEM.^29^ To mitigate these effects, two data acquisition strategies were employed, namely, sub-pixel scanning and multipass spectrum imaging. In sub-pixel scanning, the focused electron probe is continuously scanned within each pixel area and the data from the “sub-pixels” is binned into one pixel of the spectrum image.^30^ By sub-pixel scanning the electron dose is averaged over the entire pixel rather than being concentrated in one point within the pixel (assuming the probe diameter is less than the pixel size) and allows a higher pixel dwell-time without damaging the sample. Multipass spectrum imaging allows live monitoring of the sample and electron dose fractionation.^31^ It allows the examination of individual frames, ensuring that only those collected before the onset of electron damage remain for integration and maximizing the electron dose tolerance of the materials. This approach is highly effective when a direct electron detector is available, as the noise associated with photon conversion, gain, and read-out are eliminated, and the spectra are only affected by the shot noise. Consequently, multiple spectrum image frames can be summed without sacrificing the signal-to-noise ratio (SNR).

In this research, a comprehensive EELS analysis of HA and OCP was conducted. The electron beam damage of HA and OCP was extensively investigated, and a hypothesis for the damage mechanism in apatite crystals was proposed. Low-loss and core-loss spectra of P L_2,3_-edge, Ca L_2,3_-edge, and O K-edge were studied. Among these spectra, the difference in O K-edge spectra was consistent with the EELS simulation result and further used as an indicator to distinguish between HA and OCP. The EELS quantification of the Ca/P ratio was demonstrated as another route to differentiate HA and OCP. The quantification result was shown as an overlay of elemental maps of Ca and P, indicating the ability of EELS to define the boundaries of these two mineral types. Last, with the established EELS data, mouse tooth enamel was analyzed and the results were consistent with previous research results.

## 2. Materials and methods

### 2.1. Preparation of HA and OCP crystals sample

The HA and OCP single crystalline samples were prepared as reference materials for EELS analysis. The HA particles were sourced from Sigma-Aldrich, while the OCP crystals were synthesized using the precipitation method.^32,33^ This synthesis was achieved by adding 0.02 M Ca(CH_3_COO)_2_ solution dropwise to an equal volume of 0.02 M NaH_2_PO_4_ with continuous stirring at 60 °C and pH 6. After a two-hour reaction, cloudy precipitates gradually formed. The product was filtered, washed with distilled water, and dried at 37 °C to yield white OCP crystal.

### 2.2. XRD acquisition

XRD measurements were conducted using a Rigaku Miniflex (Rigaku Holdings Corporation, Japan) with Cu Kα radiation (*λ* = 1.54 Å). The X-ray voltage and current were set to 40 kV and 15 mA. A range of diffraction angles from 3° to 70° was scanned with a step size of 0.02°. Minerals were identified by comparing the resulting peaks to those in the International Center for Diffraction Data (ICDD) database and the Inorganic Crystal Structure Database (ICSD).

### 2.3. Mouse incisor sample preparation for microscopy

The preparation detail of mouse incisor samples was described in previous research.^34^ Briefly, seven-week-old wild-type and *Mmp20*^−/−^ mice were anesthetized and perfused *via* the heart with 2.5% glutaraldehyde in 0.08 M sodium cacodylate buffer containing 0.05% calcium chloride. The mandibles were dissected, and the soft tissue and labial bone were removed. Post-fixation in the same perfusion solution was carried out for 4 to 6 hours at 4 °C. The samples were washed overnight with 0.1 M sodium cacodylate buffer. Subsequently, the samples were stained with 1% reduced osmium tetroxide for 2 hours and washed several times with distilled water. The hemi-mandibles were dehydrated with an acetone gradient, embedded in epoxy resin, cross-sectioned to a thickness of 1 mm, and glued onto plastic stubs.

### 2.4. TEM sample preparation

Small amounts of reference HA and OCP powders were put in ethanol and sonicated for 10 minutes to disperse particles. 3 μl sample solutions were dropped onto carbon-coated TEM grids and dried at room temperature.

The mouse incisor TEM samples were prepared using the dual-beam FIB-SEM (Helios NanoLab™ 600, Thermo Fisher, U.S.) using the lift-out technique as described by Giannuzzi *et al.* and Jantou *et al.*^35,36^ In brief, the embedded tooth stubs were mounted on SEM stubs with carbon tape and silver glue to enhance conductivity. To further mitigate charging and drifting, a gold coating was applied to the sample stubs before placing them into the FIB-SEM chamber. Additionally, 1 μm platinum (Pt) layer was deposited onto the selected region at 30 kV and 0.28 nA to protect it from ion-milling damage. A beam current of 2.8 nA was used to create trenches around the sample. Following this, the sample was tilted to an angle of 7° for under-cut and side cuts. It was then attached to an Omniprobe and transferred to the FIB lift-out grid with the connections on the short sides of the sample using a Pt strap. The final polishing began with 30 kV and 0.28 nA initially and was reduced stepwise to 100 pA and 50 pA, finishing at 5 kV and 50 pA. The final thickness of the FIB foil was less than 100 nm to ensure optimal EELS quality.

Before placing the samples into the TEM chamber, plasma surface cleaning was processed to remove contamination and hydrocarbon deposition. The reference specimens and the FIB foils were plasma-cleaned for 25 seconds and 120 seconds, respectively.

### 2.5. STEM and EELS

The STEM and EELS investigations were conducted using Titan™^3^ 60–300 (Thermo Fisher, U.S.) at 300 kV with a beam current of 0.03 nA. EELS data were acquired with a 2.5 mm entrance aperture to achieve a collection angle to convergence angle ratio of 1.8. The monochromator was excited to improve spectral resolution, resulting in a full width at half maximum (FWHM) of the zero-loss peak (ZLP) of approximately 0.25 eV. A dispersion of 0.025 eV per channel was used to resolve spectral features for energy-loss near-edge structure (ELNES) analysis, while a dispersion of 0.1 eV per channel was selected to broaden the energy field of view (FOV) for elemental quantification and mapping. Two detectors were employed in this study: the UltraScan® (US) 1000 charge-coupled device (CCD) detector (Gatan, U.S.) and the K2™ direct electron detector (Gatan, U.S.).

The 16 × 16 sub-pixel scanning and the multi-pass imaging were used to mitigate the electron beam damage. Sub-pixel scanning distributes a uniform dose across the entire pixel rather than concentrating at the center or corner of the pixel (assuming the probe diameter is less than the pixel size), enabling a longer pixel dwell time without increasing sample damage, while multipass scanning allows the examination of individual frames, ensuring that only those collected before the onset of electron damage remain for integration and maximizing the electron dose tolerance of the materials.

The FEFF 9.6 program was used for all EELS simulations presented in this study. Several input parameters are available in FEFF calculation, including the type of exchange potential, the radius for full multiple scattering (FMS) calculation, the cluster radius for self-consistent field (SCF) potential calculations, the core-hole effect, the amplitude reduction factor, and the overlap of muffin-tin radius. Detailed descriptions of these parameters can be found in the FEFF user guide and publications by Dr John J. Rehr.^37^ The input structure files for HA and OCP were either manually constructed or generated from the WebAtoms website (now called “Larixite: Crystal Structures for X-ray Absorption Spectroscopy”, https://millenia.cars.aps.anl.gov/webatoms/).^38^

For our simulations, the Hedin–Lundqvist model was employed for exchange potential calculation. The amplitude reduction factor was set to 1.0. The radii for the FMS and SCF clusters were chosen based on the coordinates of the atoms of the system, taking approximately 100 atoms for FMS and 50 atoms into account for SCF calculations.

### 2.6. Relative quantification of Ca/P ratio

The absolute elemental quantification using EELS is possible but challenging due to the difficulty in accurately determining the inelastic cross-section. This cross-section varies not only between elements but also with the chemical environment. Also, it can be influenced by different experimental settings and analysis processes.^23^ The errors in the theoretical cross-section ultimately limit the reliability of the absolute quantification. Nevertheless, by maintaining consistent experimental conditions, these variations are largely controlled in relative quantification. Consequently, the formula for Ca/P quantification can be simplified as follows:^39^
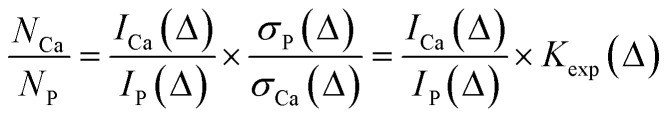
where *N* represents the number of atoms per unit area, *I* denote the intensity of the core-loss spectrum, *σ* is the partial cross-section for energy loss, and (*Δ*) indicates the energy range of the integration windows. In this research, the cross-section ratio was substituted by the experimental factor, *K*_exp_. With the known Ca/P ratio of HA and OCP, the *K*_exp_ can be determined, and the Ca/P ratio can be obtained by multiplying the intensity ratio by the experimental factor *K*_exp_.

A two-tailed Student's *t*-test was performed to evaluate whether the Ca/P ratio could effectively differentiate between material groups. The significance level (*α*) was set at 0.05, corresponding to a 95% confidence level. Probability (*p*) values were calculated using Microsoft Excel. A *p*-value less than 0.05 was considered statistically significant, indicating strong evidence (≥95% confidence) that the two groups exhibit a meaningful difference in their Ca/P ratios.

## 3. Results and discussion

### 3.1. XRD and morphology

**Fig. 1:**
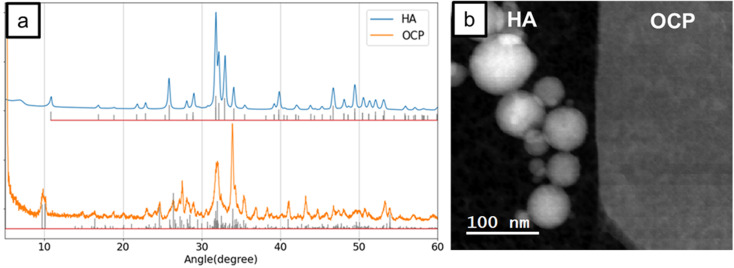
(a) The XRD patterns of HA and OCP. The intensities labeled below HA and OCP patterns were the data from ICDD (PDF# 98-000-0251) and ICSD (PDF# 04-013-3883), respectively. (b) The STEM image of spherical HA particles and OCP plate crystal.

Fig. 1a shows the XRD results for HA and OCP particles. The intensity values beneath the HA and OCP patterns correspond to reference data from ICDD (PDF# 98-000-0251) and ICSD (PDF# 04-013-3883), respectively. The XRD patterns of both HA and OCP are in excellent agreement with the reference patterns, validating the presence of pure HA and OCP particles. High-purity standards are essential for establishing a reliable EELS database for material identification.

The morphology of HA and OCP particles can be seen in Fig. 1b. HA particles are spherical nanoparticles, while OCP exhibits large plate-like crystals. The distinct morphologies of these reference materials help the acquisition of the corresponding experimental results. Although the shape of the HA reference differs from natural HA crystals, which are more typically ribbon- or needle-like, EELS analysis is primarily concerned with the chemical structure and electronic transition of materials. EELS provides detailed information about the chemical composition, bonding environment, and electronic structure of specimens.^40,41^ Energy-loss occurs when an incident electron transfers and excites an electron in an occupied state to an unoccupied energy level, losing the corresponding quantum of energy.^40^ For example, when an electron from the K-shell (1s orbital) is excited to a p-like unoccupied level, generating a characteristic signal known as the K-edge. Since binding energies are different for elements and the unoccupied energy-levels depend on the nature of the chemical bonds, the energy-loss spectrum reveals the chemical structure of materials.

Therefore, the impact of crystal morphology on EELS analysis is negligible in this research.

### 3.2. Evaluation of electron beam damage

**Fig. 2:**
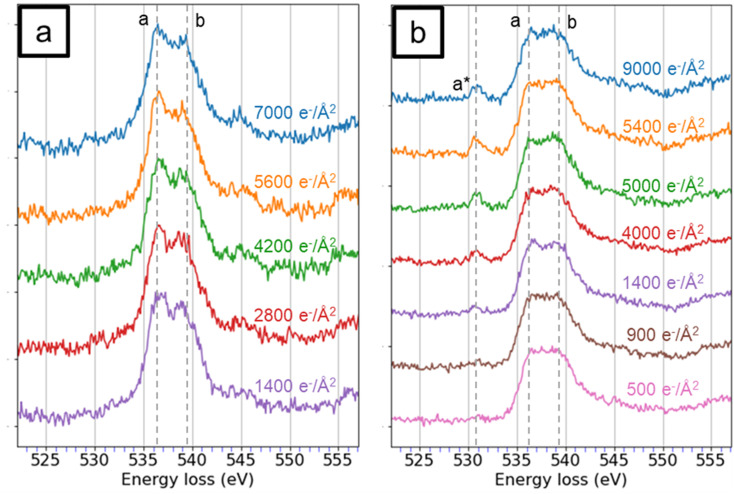
The oxygen K-edge spectra of (a) HA and (b) OCP with various amounts of cumulative electron dose. The peaks a*, a, and b were at 530.7 eV, 536.8 eV, and 539.5 eV, respectively.

The electron beam damage on HA and OCP was evaluated through a series of oxygen K-edge spectra. Multipass imaging and drift correction were applied to accumulate electron doses in the selected area. Fig. 2 illustrates the changes in the O K-edge spectra of HA and OCP. In Fig. 2a, at an electron dose of 1400 e^−^ per Å^2^, two peaks (a) and (b), 536.8 eV and 539.5 eV, respectively, with a noticeable dip between them are observed. As the cumulative electron dose increased, the main peaks remained relatively stable, but the dip between the peaks gradually flattened.

In Fig. 2b, the ELNES of OCP in O K-edge spectra is observed to differ from those of HA. The dip between peaks (a) and (b) was barely visible at 500 and 900 e^−^ per Å^2^. As the electron dose increased, a new peak (a*) around 530.7 eV emerged and intensified. This peak is attributed to the formation of radical oxygen species and indicates the electron beam damage.^42^ These findings suggested that OCP is more sensitive to the exposure of electron beam and has a lower critical dose of damage than HA. The difference in beam resistance is likely related to the chemical structure of OCP (Ca_8_(HPO_4_)_2_(PO_4_)_4_). In OCP, oxygen atoms are present not only in the phosphate group (PO_4_^3−^) but also in the hydrogen phosphate group (HPO_4_^2−^). The O–H bonds in the hydrogen phosphate group are cleaved more readily under electron beam exposure, leading to the generation of radical oxygen species and the appearance of the pre-peak signal in Fig. 2b. In contrast, HA lacks hydrogen phosphate, resulting in higher resistance to electron beam damage. As the pre-peak (a*) is recognized as an indicator of electron beam damage, Fig. 2 shows that the O K-edge spectra of OCP exhibit a barely detectable pre-peak at a cumulative dose of 900 e^−^ per Å^2^, while the pre-peak becomes visible after 1400 e^−^ per Å^2^. This suggests that the critical dose of OCP specimen is approximately 900 e^−^ per Å^2^. Consequently, the electron dose of approximately 500 e^−^ per Å^2^ was maintained to minimize damage on OCP. Regarding to HA, although the EELS analysis demonstrates that HA has better resistance to radiation damage compared to OCP, an electron dose of approximately 500 e^−^ per Å^2^ was used for consistency.

### 3.3. EELS analysis of reference materials

The EEL spectrum can be divided into low-loss regions and high-loss (core-loss) regions. The low-loss spectrum typically spans energy losses less than 50 eV and is associated with phonon excitations, plasmon resonance, and the low-energy outer shell ionization edges.^22^ The high-loss spectrum corresponds to energy losses greater than 50 eV and originates from the inner shell ionization and electronic transition between orbitals. In this study, we will analyze and discuss the low-loss region as well as the P L_2,3_-edge, Ca L_2,3_-edge, and O K-edge spectra of HA and OCP.

#### 3.3.1. Low-loss spectra

**Fig. 3:**
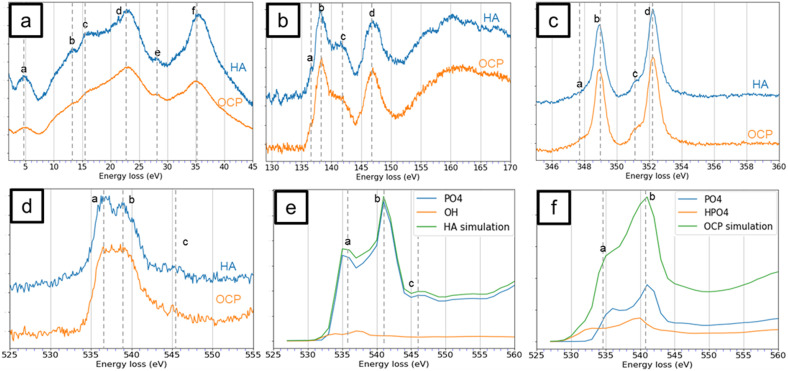
The EEL spectra of HA and OCP in different energy-loss regions, including (a) the low-loss region. (b) The P L_2,3_-edge, (c) the Ca L_2,3_-edge, and (d) the O K-edge. The P L_2,3_-edge and Ca L_2,3_-edge spectra from HA and OCP were identical while the O K-edge spectra from them were different. The EELS simulation of the O K-edge spectra of (e) HA and (f) OCP were calculated with FEFF software. The spectra of O in different functional groups were simulated individually. The simulation results of HA and OCP are the addition of spectra from each functional group according to their portion in the material.

The low-loss spectra of both HA and OCP reveal six distinct peaks as shown in Fig. 3a. Peak (a) located at 5 eV is associated with a π* plasmon resonance. Peaks (b) and (c) at approximately 13 eV and 15 eV are likely associated with the features in the imaginary part of the complex dielectric function. Peak (d) is observed at 23 eV and corresponds to the volume plasmon excitation.^23,43^ Peaks (e) and (f) are at 28 eV and 35 eV and attributed to the Ca M_2,3_-edge. Although the relative intensity and spectral shape exhibit slight differences, distinguishing between HA and OCP based solely on these features is challenging. The relative intensity and spectral shape of low-loss spectra are difficult to quantify as their features are easily influenced by experimental variations, such as background subtraction and sample thickness. Therefore, the low-loss spectra are not a reliable approach to differentiate HA and OCP.

#### 3.3.2. P L_2,3_-edge

In Fig. 3b, the P L_2,3_-edge of high-loss spectra for both HA and OCP display four distinct peaks at 136.5 eV, 138.5 eV, 141.5 eV, and 147 eV. Because of the similar electron structure of PO_4_^3−^ and SiO_4_^4−^, the molecular orbital (MO) calculation for PO_4_^3−^ can be referenced from SiO_4_^4−^. According to Tossel's MO calculations, the lowest unoccupied molecular orbitals are labeled 6t_2_, 6a_1_, and 2e.^44^ However, the core-hole effect pulls the energy level of 6a_1_ down below 6t_2_, which was verified by McComb *et al.*^45^ Consequently, peaks (a), (b), and (d) are attributed to the 6a_1_, 6t_2_, and 2e orbitals, respectively. Peak (c) is not assigned to any MOs as no available state exists between 6t_2_ and 2e and is normally attributed to a multiple scattering effect.^46^ Despite these insights, the differences in ELNES of phosphorus between the HA and OCP are not significant enough to distinguish between them.

#### 3.3.3. Ca L_2,3_-edge

HA and OCP spectra revealed two intense sharp peaks (b) and (d) along with two weaker shoulders (a) and (c) in Fig. 3c. Peaks (a) and (b) correspond to the L_3_ edge, and the peaks (c) and (d) are related to the L_2_ edge. The energy separation of the L_3_ and L_2_ edges is due to the spin–orbit splitting of calcium, which is approximately 3.3 eV.^47^ The small shoulder peaks (a) and (c) are attributed to the crystal field splitting effect.^48^ In symmetrical coordination environments, d-orbitals (d_*xy*_, d_*xz*_, d_*yz*_, d_*x*^2^−*y*^2^_, d_*z*^2^_) would split into two levels by a crystal field splitting parameter.^23^ Consequently, the appearance of peaks (b) and (c) confirm the symmetrical coordination of calcium in HA and OCP. The separation between peak (a) and (b), and between (c) and (d) are approximately 1 eV, consistent with the findings reported by Naftel *et al.*^48^ Again, the difference between HA and OCP spectra is minimal.

#### 3.3.4. O K-edge

The O K-edge spectra of HA and OCP are shown in Fig. 3d. Unlike the P L_2,3_-edge and Ca L_2,3_-edge spectra, distinct differences between HA and OCP spectra are observed. The HA spectrum displays two prominent peaks at 536.8 eV and 539.5 eV, followed by a small feature at 545.4 eV. In contrast, the OCP spectrum shows only one broad peak where the dip between 536.8 eV and 539.5 eV is not distinct, and the feature at 545.4 eV is less pronounced. These differences in ELNES features are associated with variations in the chemical environment of oxygen between HA and OCP. To further investigate the spectral difference and validate the findings, EELS simulations of HA and OCP were conducted using FEFF software, as shown in Fig. 3e and f. FEFF simulates EELS using a multiple scattering approach and is highly effective.^37,49^ Although the simulated spectrum may exhibit shifting or broadening due to the experimental factors not included in the simulations, the shape of the ELNES, relative intensity, and trends are considered reliable.

In HA, 24 oxygen atoms are bonded with phosphorus to form six tetrahedral phosphate groups (PO_4_^3−^), while 2 oxygen atoms are bonded to hydrogen, forming two hydroxide groups (OH^−^). In contrast, OCP contains 16 oxygen atoms coordinated with phosphorus to form four tetrahedral phosphate groups, and 8 additional oxygen atoms also bond to phosphorus, but among them, two oxygen atoms bond with hydrogen, forming two hydrogen phosphate groups (HPO_4_^2−^).

FEFF was used to individually calculate the oxygen K-edge spectra for all 26 oxygen atoms in HA and 24 oxygen atoms in OCP. The spectra originating from the same functional groups were summed to represent the spectrum of each corresponding functional group. The final HA and OCP simulation spectra are the integration of all spectra from the corresponding functional groups, as shown in Fig. 3e and f. It is observed that the PO_4_^3−^ groups in both HA and OCP exhibit two peaks at approximately 536 eV and 541 eV. The OH^−^ group shows two peaks at approximately 534 eV and 537.5, while the HPO_4_^2−^ group displays peaks at approximately 532.5 eV and 540 eV.

In HA, the majority of oxygen atoms are associated with the phosphate group (PO_4_^3−^), with only a small portion associated with the hydroxide group (OH^−^). Therefore, the O K-edge simulation of HA is primarily dominated by the PO_4_^3−^ group, exhibiting two peaks at approximately 536 eV and 541 eV. In contrast, OCP contains over half of the oxygen atoms from the phosphate group (PO_4_^3−^), and one-third oxygen atoms from the hydrogen phosphate group (HPO_4_^2−^). Consequently, the addition of the HPO_4_^2−^ spectrum fills the dip observed in the PO_4_^3−^, leading to a broad feature with no distinct minimum in the OCP simulation. In summary, due to the presence of the hydrogen phosphate group (HPO_4_^2−^) in OCP leads to different characteristic features in O K-edge spectra between HA and OCP.

Although there are slight disagreements in peak positions between the simulated and experimental spectra, the near-edge structures in both simulations and experimental data are consistent. The disagreements between the simulated and experimental spectra might arise from several possible factors. For instance, the zero in energy simulation is referred to as the muffin tin potential <0, and is not aligned with the vacuum level of various compounds, resulting in energy shifts expected in the simulated results.^50^ These distinctions can serve as an indicator for further identification of HA and OCP.

The EELS simulation results also provide insight into the electron beam damage mechanism of OCP and HA. As shown in Fig. 2b, a dip between peaks (a) and (b) is observed in OCP with the cumulative dose range from 1400 to 5000 e^−^ per Å^2^, becoming similar to HA. However, the dip became barely visible when the cumulative dose was beyond 5400 e^−^ per Å^2^, and eventually disappeared even as the dose increased to 9000 e^−^ per Å^2^. This behavior is likely related to the presence of hydrogen phosphate groups in the OCP. The breaking of O–H bonds in HPO_4_^2−^ not only generated radical oxygens but also converted HPO_4_^2−^ into PO_4_^3−^. Once this transformation occurs in OCP, the dip between peaks will no longer be filled, and the ELENS of OCP will be similar to HA. Consequently, when damage peak (a*) is observed, the O K-edge spectrum of OCP evolves toward the two-peak feature similar to that of HA. With the increase of electron dose, the dip becomes flatter in both HA and OCP spectra. The EELS simulation and experimental results provide an atomic-level explanation for the phase transformation of OCP to HA under electron beam exposure.

### 3.4. Relative quantification of Ca/P ratio

In general, the experimental factor *K*_exp_ varies between different materials due to differences in energy-loss cross-section. However, because of the similarity in P L_2,3_-edge and Ca L_2,3_-edge of HA and OCP, their *K*_exp_ value is expected to be comparable. The experimental factor (*K*_exp_) for the relative quantification of the Ca/P ratio was determined using pure HA nanoparticles. A total of 11 EELS acquisitions were collected, and the results are summarized in the ESI.† Using the known Ca/P ratio of HA, 1.67, the *K*_exp_ was calibrated to be 1.83 ± 0.08 for this research. To confirm the reliability of the *K*_exp_, the Ca/P ratio of OCP was calculated and yielded a value of 1.36 ± 0.05, which is close to the theoretical ratio of 1.33 for OCP. The Ca/P ratio of HA was calculated and yielded a value of 1.67 ± 0.07. A two-tailed Student's *t*-test was conducted between the HA and OCP datasets, yielding a statistically significant difference (*p* = 2.41 × 10^−5^). The datasets used for the calculation of Ca/P ratio and *p*-value are presented in Tables S.2 and S.3. The result confirms the reliability of *K*_exp_ for the relative quantification in these materials.

### 3.5. Elemental mapping of HA and OCP

**Fig. 4:**
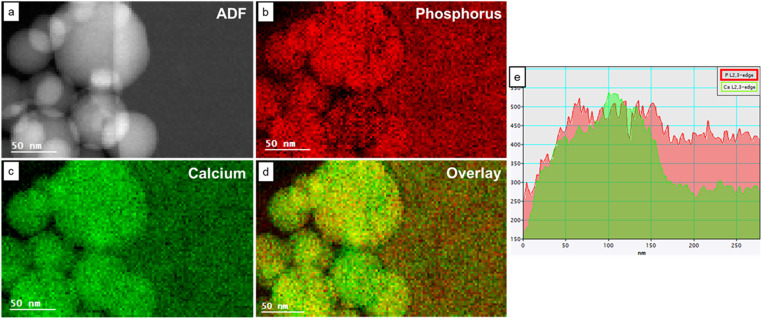
(a) The STEM Annular Dark Field (ADF) image and the corresponding eels elemental mapping of (b) the P L_2,3_-edge and (c) the Ca L_2,3_-edge. (d) The overlay of phosphorus map and calcium map. The red color represents the phosphorus signal, and the green color represents the calcium signal. (e) The intensity projection of P and Ca signal to the *x*-axis. The *x*-axis represents the distance from left to right, and the *y*-axis indicates the intensities of P and Ca.

Fig. 4 shows the STEM ADF image and the elemental maps of Ca and P in an area containing both HA and OCP reference materials. In the P maps, the intensities of HA and OCP were similar. However, in the Ca maps, the HA particles showed higher intensity than the OCP crystal. Fig. 4e shows the intensity projection of the Ca and P signal onto the *x*-axis, revealing different Ca/P intensity ratios and the boundary between HA and OCP. The ratio was higher on the left side (HA particles) and lower on the right side (OCP crystal). Moreover, the Ca/P ratio can be calculated by simply multiplying the Ca/P intensity ratio by the calibrated factor *K*_exp_.

### 3.6. Dental enamel from WT and KO mouse incisors

**Fig. 5:**
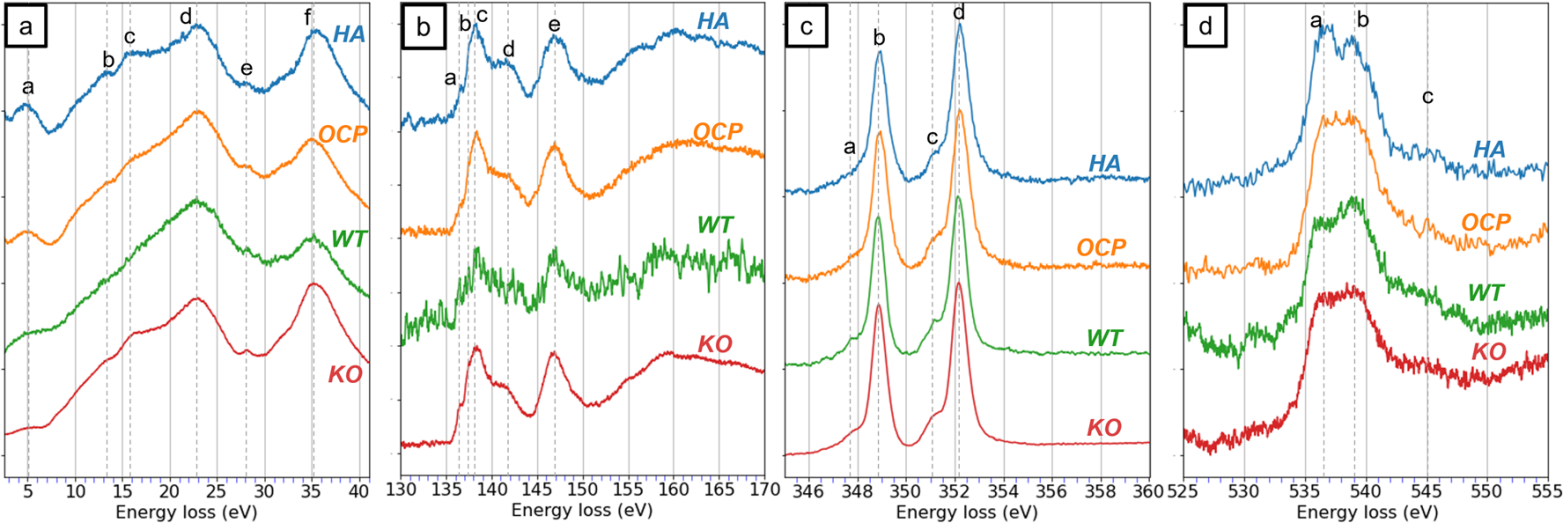
The EEL spectra from HA, OCP, wild-type (WT) enamel, and *Mmp20* knockout (KO) enamel in different energy-loss regions, including (a) the low-loss region. (b) The P L_2,3_-edge, (c) the Ca L_2,3_-edge, and (d) the O K-edge. The WT and KO spectra were acquired from the regions indicated in Fig. 6(a) and (d).

**Fig. 6:**
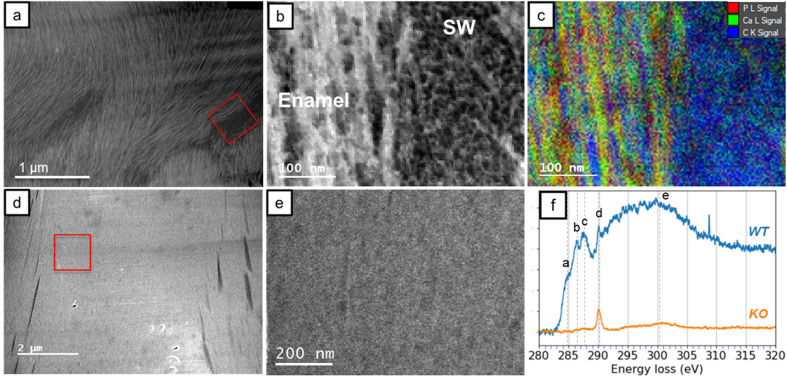
(a) The STEM ADF images of WT enamel in the secretory stage. The red box indicates where the WT spectra were acquired. SW, Space of Weber. (b) The STEM ADF image of the region indicated by the red box in (a). (c) Shows the corresponding elemental mapping of (b). The red, green, and blue colors indicate the P L_2,3_-edge, Ca L_2,3_-edge, and C K-edge signals, respectively. (d) The STEM ADF images of KO fan-shaped enamel in the secretory stage. The red box indicates where the KO spectra were acquired. (e) The STEM ADF image of the red box in (d). (f) The C K-edge spectra of WT and KO enamel.

With the established EELS results from HA and OCP, the materials in WT and KO enamel were investigated. The WT and KO specimens were extracted from teeth in the secretory stage of development. At this stage, the WT enamel was not fully mineralized and consisted of enamel crystals and enamel matrix. On the other hand, the fan structure KO enamel was highly crystallized and presented a single crystal diffraction pattern.^14^ The EELS data are presented in Fig. 5, and the areas selected for the EELS acquisition are shown in Fig. 6. Notably, in the secretory WT enamel, there are regions that lack enamel crystals, known as the Space of Weber (SW).^51^

In the low-loss spectra (Fig. 5a), six peaks are identified in the KO spectrum, whereas some fine features, such as peaks (b) and (c), are less distinct in the WT spectrum. This difference may be attributed to the complex environment of the WT specimen. Since WT enamel is a mixture of crystal and proteins, peaks (b) and (c) became subtle in the spectra, leading to different shapes of the spectrum. In contrast, the KO enamel being a single crystal showed distinct features in the low-loss spectrum and matched the peaks from reference materials. Regardless, the low-loss features are insufficient to identify the materials of KO enamel.

In the P L_2,3_-edge, peaks in KO enamel are distinctly recognizable and align well with reference spectra. For the WT enamel, although the P signal is noisier, most of the major peaks remain identifiable. In the Ca L_2,3_-edge, minimal differences are observed. All spectra from HA, OCP, WT, and KO samples appear nearly identical. Even though the comparison of the low-loss, P L_2,3_-edge, and Ca L_2,3_-edge does not effectively identify whether the WT and KO are HA or OCP, the similarities indicate that the materials in both WT and KO consisted of components with similar chemical structures as HA and OCP. This observation was consistent with our previous research.^14^

The O K-edge spectrum is a key indicator for distinguishing between HA and OCP. In the O K-edge spectrum of WT, despite the noisier signals, a peak at 536 eV and a more intense peak at 539 eV were observed. In contrast, only a broader peak was detected in the KO spectrum. Acquired under the same spectral resolution, the characteristic feature of HA was not observed in the KO spectrum. This result supports the hypothesis that the WT enamel primarily consisted of HA while the KO enamel was composed of OCP.

Fig. 6 shows the images of the WT and KO enamel, and the red squares indicate the areas where the EELS data were acquired. The STEM ADF image and the elemental mapping of WT enamel are presented in Fig. 6b and c. In the ADF image, we can observe the shape and texture of the enamel crystals on the left side and identify the SW region on the right side. In the EELS mapping, the outlines of the fibrous enamel crystals were well defined, and the gaps between individual crystals were also detected. The elemental map indicates that the SW region contained no mineral crystals but comprised organic matter, possibly proteins. The relative quantification was exclusively processed on crystal regions, and the Ca/P ratio of WT enamels was approximately 1.81 ± 0.04. This result is higher than the Ca/P ratio of HA, which is 1.66. One possible reason is the substitution of the phosphate groups with carbonate groups. Previously published research has suggested that immature enamel may consist of carbonate hydroxyapatite instead of pure HA.^27,52,53^ In carbonate HA, the Ca/P ratio is increased due to the reduction of the phosphate group.

Compared to the WT enamel, KO enamel did not show fiber crystals, instead appearing as single crystal platelets. The elemental map of KO enamel is not shown as it is uniform with few features. The relative quantification of the Ca/P ratio for KO enamel is 1.38 ± 0.07, lower than that of WT enamel. The two-tailed Student's *t*-test was conducted to compare the Ca/P ratios between WT and KO enamel datasets (Tables S.4 and S.5), as well as between OCP and KO enamel datasets, yielding a *p*-value of 1.17 × 10^−4^ and 0.75, respectively. These results indicate a statistically significant difference between KO and WT enamel compositions, while no significant difference was observed between KO enamel and OCP. This finding supports the hypothesis that the KO enamel is predominantly composed of OCP rather than HA. It needs to be mentioned that the Ca/P ratio of KO was slightly higher than that of pure OCP. Similar to WT enamel, the substitution by the carbonate group could be a factor.

Fig. 6f reveals the EELS investigation of the carbon K-edge. Four sharp peaks and one broad peak (e) were labeled in the WT spectrum, while only one intense peak (d) and a weak feature (e) were observed in the KO enamel. The identification of peaks in the C K-edge is well estimated.^24,54–57^ Peak (a) at 285 eV corresponds to the transition from 1s to π*(C=C). Peak (b) and (c), located at 286.5 and 288 eV, are attributed to π*(C=N) and the π*(C=O), respectively. Peak (d) at 290 eV is associated with a transition involving the π*(C=O) of the carbonate minerals. The last broad peak (e) is correlated to the transition to σ*(C–C) state associated with single bonds. The presence of peak (d) supported the hypothesis that carbonate is present in both WT and KO enamel. The remaining features in the C K-edge of WT indicate the presence of other organic matter, such as proteins.

## 4. Conclusions

This study reports a strategy to distinguish HA and OCP using EELS analysis and shows its application to identify the enamel materials from WT and *Mmp20* KO mice. The EELS analysis focuses on the investigation of the region of low-loss, P L_2,3_-edge, Ca L_2,3_-edge, and O K-edge. The features in low-loss spectra, P L_2,3_-edge, and Ca L_2,3_-edge are consistent with the chemical structure of HA and OCP and have no distinctive difference between the HA and OCP spectra from these regions. However, the presence of the hydroxide and hydrogen phosphate groups in HA and OCP brings insights into the differentiation. The O K-edge spectrum of HA showed two characteristic peaks, but that of OCP only showed a broad peak. The EELS simulations conducted using the FEFF software revealed consistent patterns in the experimental data. Consequently, the O K-edge spectrum was validated as a reliable indicator for distinguishing between HA and OCP.

The evaluation of electron beam damage on HA and OCP crystals highlighted the electron dose limit for this study and provided an explanation of the electron beam damage mechanism in apatite. OCP is more sensitive to electron beam exposure than HA and is attributed to the presence of H–O bonds in the hydrogen phosphate group. The breakdown of the hydrogen phosphate group in OCP increased the relative abundance of the phosphate group, causing the O K-edge spectra of OCP to present the two-peak feature similar to that of HA.

The relative quantification of Ca/P ratio and the elemental mapping are the second indicators to differentiate HA and OCP. With the known Ca/P ratio of HA, the *K*_exp_ factor was calculated to enable the relative quantification in this study. The Ca/P ratio was determined by multiplying the intensity ratio by the *K*_exp_ factor. The calculated ratio of OCP is 1.36 ± 0.05, which is close to the theoretical ratio of 1.33, while the ratio of HA is 1.67 ± 0.07, which is close to the theoretical ratio of 1.67. The Student's *t*-test results verify the statistical significance between these two datasets. The elemental mapping not only well outlines the shapes and boundaries of HA and OCP but also provides an intensity ratio for quantification.

With the indicator of O K-edge, the enamel material from WT exhibited a characteristic feature of HA, while the KO enamel showed OCP patterns. Additionally, the Ca/P ratio of WT and KO enamel are distinct, measured at 1.81 ± 0.04 and 1.38 ± 0.07, respectively. Both Ca/P ratio from WT and KO were higher than the pure HA and OCP. The discrepancy is possibly associated with the substitution of the phosphate group by the carbonated group. This hypothesis is supported by the detection of carbonate in carbon K-edge.

With the EELS technique, a comprehensive analysis including chemical structure, elemental composition, and damage mechanism was carried out in this research. Additionally, elemental mapping with great spatial resolution was also revealed. This research demonstrates the potential of EELS and provides additional functionalities for EELS analysis on materials.

## Data availability

Crystallographic data for hydroxyapatite and octacalcium phosphate have been deposited at the International Center for Diffraction Data database (PDF# 98-000-0251) and the Inorganic Crystal Structure Database (PDF# 04-013-3883), respectively. The EELS data was analyzed with the DigitalMicrograph Software announced by Gatan. The spectra simulation was processed with FEFF 9 software.^37^ Electron microscopy was performed at the Center for Electron Microscopy and Analysis (CEMAS) at The Ohio State University.

## Author contributions

Y. H. Hsu: investigation, writing – original draft. A. Hassan: resources. A. Trout: methodology. J. D. Bartlett: funding acquisition, supervision, writing – review & editing. C. E. Smith: resources, writing – review & editing. D. W. McComb: funding acquisition, supervision, writing – review & editing.

## Conflicts of interest

There are no conflicts of interest to declare.

## Supplementary Material

RA-015-D4RA08124B-s001
