# Supplementary material for: Identification and elemental mapping of enamel minerals with electron energy-loss spectroscopy
Source: RSC Adv. 2025 May 7;15(19):14848–58. doi: 10.1039/d4ra08124b (PMC12057556; doi:10.1039/d4ra08124b)
Supplement: RA-015-D4RA08124B-s001 [file RA-015-D4RA08124B-s001.pdf]

Supplementary Information (SI) for RSC Advances.
This journal is © The Royal Society of Chemistry 2025

Supplement information

The experimental factor (K_{exp}) for the relative quantification of the Ca/P ratio was determined using pure HA nanoparticles. A total of 11 EELS acquisitions were collected, as shown in Figure S.1, and the results are summarized in Table S.1.

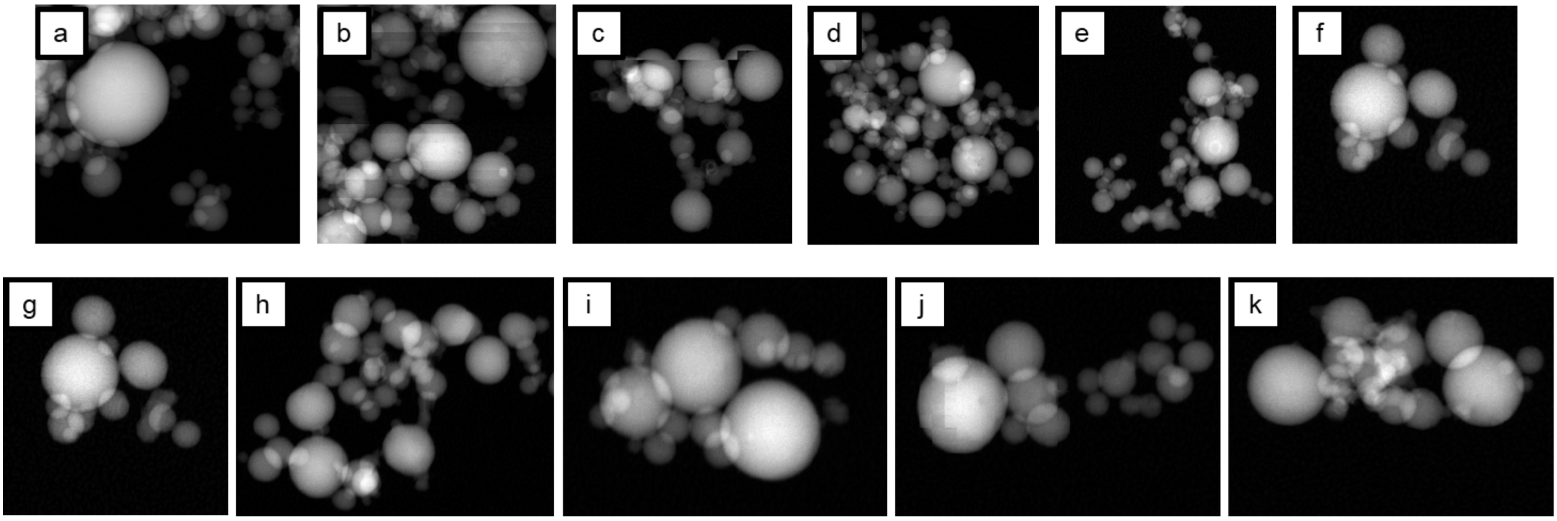

Figure S.1 STEM images of pure HA nanoparticles were acquired and accompanied by the collection of EEL spectra.

Regions	Intensity (Ca)	Intensity (P)	Ca/P ratio	K_{exp}
a	4.47E+06	4.51E+06	1.667	1.68
b	3.12E+06	3.33E+06	1.667	1.78
c	1.43E+06	1.55E+06	1.667	1.81
d	3.66E+06	4.28E+06	1.667	1.95
e	1.43E+06	1.60E+06	1.667	1.87
f	4.64E+05	5.01E+05	1.667	1.8
g	3.00E+06	3.32E+06	1.667	1.84
h	1.52E+06	1.59E+06	1.667	1.74
i	7.70E+05	8.69E+05	1.667	1.88
j	1.51E+06	1.68E+06	1.667	1.85
k	1.50E+06	1.75E+06	1.667	1.95
**Average**				1.83
**STD**				0.08

Table S.1. Summarized EELS analyses for the calibration of the experimental factor (K_{exp}). STD: standard deviation.

The tables used for the calculation of Ca/P ratio and the values of Student's t-test value

Regions (HA)	Intensity (Ca)	Intensity (P)	K_{exp}	Ca/P ratio
a	4.47E+06	4.51E+06	1.83	1.81
b	3.12E+06	3.33E+06	1.83	1.71
c	1.43E+06	1.55E+06	1.83	1.69
d	3.66E+06	4.28E+06	1.83	1.56
e	1.43E+06	1.60E+06	1.83	1.64
f	4.64E+05	5.01E+05	1.83	1.69
g	3.00E+06	3.32E+06	1.83	1.65
h	1.52E+06	1.59E+06	1.83	1.75
i	7.70E+05	8.69E+05	1.83	1.62
j	1.51E+06	1.68E+06	1.83	1.64
k	1.50E+06	1.75E+06	1.83	1.57
**Avg**				1.67
**Std**				0.07

Table S.2. Summarized EELS analyses from the HA reference specimens for the calculation of Ca/P ratio. STD: standard deviation.

are presented in Tables S.2-5.

Regions (OCP)	Intensity (Ca)	Intensity (P)	K_{exp}	Ca/P ratio
a	4.39E+05	5.69E+05	1.83	1.42
b	4.41E+05	6.03E+05	1.83	1.34
c	4.34E+05	5.97E+05	1.83	1.33
Avg				1.36
Std				0.05

Table S.3. Summarized EELS analyses from the OCP reference specimens for the calculation of Ca/P ratio. STD: standard deviation.

Table S.4. Summarized EELS analyses from the WT enamel for the calculation of Ca/P ratio. STD: standard deviation.

Regions (WT)	Intensity (Ca)	Intensity (P)	K_{exp}	Ca/P ratio
a	7.12E+06	7.13E+06	1.83	1.83
b	2.55E+04	2.63E+04	1.83	1.78
Avg				1.81
Std				0.04

Regions (KO)	Intensity (Ca)	Intensity (P)	K_{exp}	Ca/P ratio
a	7.09E+06	8.99E+06	1.83	1.45
b	4.28E+06	5.47E+06	1.83	1.43
c	1.67E+07	2.33E+07	1.83	1.32
d	3.17E+07	4.21E+07	1.83	1.38
e	2.79E+07	4.11E+07	1.83	1.24
f	2.29E+07	2.94E+07	1.83	1.43
g	1.10E+07	1.46E+07	1.83	1.39
Avg				1.38
**Std**				0.07

Table S.5. Summarized EELS analyses from the KO enamel for the calculation of Ca/P ratio. STD: standard deviation.
